# Digital Design for Lower Incisor Position Correction in a Growing Patient with Mandibular Retrusion with ClinCheckÒ Software: A Case Report

**DOI:** 10.3390/jcm15103647

**Published:** 2026-05-09

**Authors:** Lupini Daniela, Caruso Sara, Cozzani Mauro, Caruso Silvia

**Affiliations:** 1 Università degli Studi di Trieste Trieste, 34127 Trieste, Italy; danielalupini@gmail.com; 2 Department of Life, Health and Environmental Sciences, University of L’Aquila, 67100 L’Aquila, Italy; 3 Istituto Giuseppe Cozzani, 19125 La Spezia, Italy

**Keywords:** digital cephalometry, mandibular advancement, lower incisor, clear aligners, orthodontic planning

## Abstract

**Background**: The majority of Class II malocclusions stem from mandibular deficiency, leading to chin retrusion. In growing patients, the ideal correction—aiming for a skeletal mandibular response—should avoid common pitfalls such as “Point B” dropping postero-inferiorly, excessive labial proclination of mandibular incisors, or the lingual tipping and extrusion of maxillary incisors. When planning mandibular advancement (MA) using clear aligners with integrated advancement features, biomechanical forces are not the only consideration; precise management of the lower incisor position is critical for success. Current literature highlights not a good control in digital planning software: these platforms are primarily dentoalveolar-based and lack integrated cephalometric analysis. Consequently, mandibular advancement is often defined by standard linear parameters (typically 2 mm per step), while incisor position is managed through virtual alignment without correlation to cephalometric landmarks like the Pogonion, NB line, or IMPA. The software cannot monitor real-time sagittal or vertical skeletal relationships, the software will elaborate the treatment planning after doctor’s prescription, the clinician must manually adjust incisor positioning based on external cephalometric analysis to prevent dental compensation or excessive proclination. **Aim:** This clinical case demonstrates a specific arch preparation protocol designed to optimize mandibular advancement in a growing patient with mandibular retrusion. **Methods**: A 12-year-old female presented with a skeletal and dental Class II malocclusion, characterized by increased overjet and a normal overbite. Treatment was conducted using Invisalign^®^ clear aligners (22 h/day wear, weekly changes). The treatment objectives were: *transverse:* Correct upper dentoalveolar contraction and coordinate arch form while restoring midline alignment; *sagittal:* establish Class I molar and canine relationships by correcting the overjet and reducing the labial inclination of the lower incisors; *vertical:* level the curve of Spee. A key clinical condition of our protocol was the pre-advancement phase: the lower arch was reshaped by reducing the buccolingual inclination (retroclination) and intruding the lower incisors. This was specifically intended to increase the available overjet space, creating the necessary room for subsequent mandibular advancement. **Results** Treatment was completed in 24 months with high patient compliance. Objectives were successfully met, including the correction of skeletal and dental discrepancies, the establishment of harmonious arch forms, and precise overjet reduction through enhanced control of the mandibular incisors. **Conclusions:** This case report outlines an optimized clinical strategy for Class II correction. *Cephalometric Integration:* Perform an initial analysis outside the digital planning software to define the ideal IMPA and NB angles. *Anatomic Verification:* Utilize radiographic overlays to ensure tooth movement remains within alveolar bone limits. *Pre-MA Optimization:* Prioritize a “pre-advancement” phase to maximize the sagittal inter-arch space (overjet). A larger overjet allows for a more significant orthopedic effect from the MA features. *Stepwise Advancement:* Implement mandibular advancement in increments (≥2 mm) with periodic clinical reassessment to facilitate the adaptation of the muscular sling and functional occlusion.

## 1. Introduction

In recent years, the widespread use of clear aligners has profoundly changed the approach to orthodontic treatment planning, extending their application to growing patients. However, the scientific literature highlights that currently available digital protocols still present diagnostic and biomechanical limitations, particularly in cases of mandibular retrusion treated with functional advancement in growing patients [1]. The majority of Class II malocclusions stem from mandibular deficiency, resulting in chin retrusion [2]. In growing patients, the ideal correction—aiming for a true skeletal response—should avoid common compensations such as the postero-inferior displacement of Point B, excessive labial proclination of the mandibular incisors, and the lingual tipping or extrusion of the maxillary incisors [3]. When planning mandibular advancement in growing patients using aligners with advancement features, we must consider not only the biomechanics of advancement, but also the positioning of the lower incisors to ensure successful treatment.

The greatest challenge is translating a careful orthodontic diagnosis, which includes planning the position of the lower incisor, into the software used. In the clinical case presented, Invisalign’s ClinCheck^®^ software (ClinCheck Pro 6.0) is used, which has a specific protocol for mandibular advancement.

For the Invisalign^®^ technique, two important aspects are considered: considerations regarding patient selection (if eligible for this treatment) and the clinical considerations for the software in creating the pre-MA phase.

About clinical considerations and eligibility: The teeth adjacent to and underlying the Precision Wings must be present and stable throughout the entire mandibular advancement phase of treatment. Fully erupted dentition, with the exception of the canines, second molars, and third molars, must be present. There must be no supernumerary teeth on the buccal side of the premolars and molars. The company recommends using the technique at the physician’s discretion in the following patients: patients with severe deep bite > 7 mm (as results depend on anterior intrusion and leveling of the curve of Spee), patients younger than 10 years of age due to deciduous dentition under the Precision Wings, and those older than 17 years of age due to minimal growth. Posterior crossbite may require crossbite improvement in the pre-MA phase to avoid interference with wing placement during mandibular advancement. Only partial resolution of the posterior crossbite may cause posterior occlusal interference manifesting as a posterior open bite.

The pre-Ma phase is automatically performed under the following conditions to ensure Precision Wing positioning: deep bite > 7 mm, molar rotation > 20 degrees, first advancement of Class II Division II incisors to achieve ovj of 2 mm, and crossbite correction to achieve a posterior ovj between −2 mm and 4 mm.

Both for the clinical conditions for patient inclusion and for the start of the Pre-Ma phase, the software considers the wearability of the Precision Wings as the primary parameter. Diagnostic parameters are not taken into account, as the diagnosis remains the responsibility of the orthodontist.

Several authors [4,5] highlight that virtual planning software allows clinicians to program mandibular displacement in predefined steps of approximately 2 mm, but these systems do not integrate internal cephalometric analysis tools, making it difficult to accurately assess skeletal relationships and the ideal position of the lower incisors. This represents an important concept to understand, since the cephalometric determination of incisor position (IMPA, NB, pogonion) is fundamental for guiding therapy in a physiological manner, respecting alveolar limits and maintaining dentofacial aesthetic harmony [6].

Evidence comparing removable functional appliances and fixed/removable alternatives in adolescents highlights variable soft-tissue and skeletal responses that should be considered when planning mandibular advancement with aligners [7].

The aim of this clinical case is to demonstrate the arch preparation protocol prior to mandibular advancement in a growing patient with mandibular retrusion.

The goals of orthodontic treatment in the transverse plane are: correction of maxillary dentoalveolar constriction, correction of left posterior crossbite, normalization of arch form, restoration of alignment between the upper and lower dental midlines, and correction of the buccal inclination of the lower incisors.

In the sagittal plane: restoration of Class I occlusal relationships, correction of overjet, and correction of the buccal inclination of the lower incisors.

In the vertical plane: leveling of the curve of Spee.

## 2. Materials and Methods

A 12-year-old female patient (S.G.) presented to our attention.

**Figure 1:**
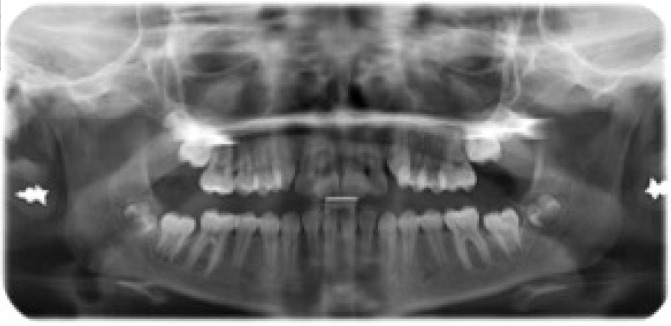
Initial panoramic x-ray.

**Figure 2:**
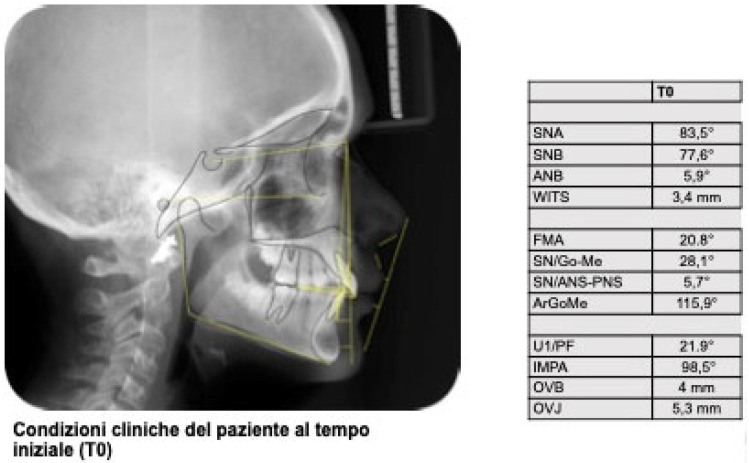
Initial teleradiography and cephalometric analysis.

Clinical examination revealed a full permanent dentition, with teeth 1.3 and 2.3 (maxillary canines) in the passive stage of eruption. To establish a precise orthodontic diagnosis, a comprehensive diagnostic assessment was performed, including a panoramic radiograph (orthopantomogram) and a lateral cephalometric radiograph. The panoramic radiograph confirmed the absence of carious lesions and indicated that periodontal health was within normal physiological limits. Notably, Molar-Incisor Hypomineralization (MIH) was observed on teeth 1.6 and 2.6 (maxillary first molars). The selection of clear aligner therapy facilitated superior management and monitoring of these MIH-affected elements; specifically, the use of fluoride-releasing resins for attachment bonding minimized potential damage to the demineralized enamel, ensuring the structural integrity of the affected teeth throughout treatment (Figure 1). Cephalometric analysis was performed by a single examiner using WebCeph software 2.0.0. The results confirmed a Skeletal Class II relationship primarily due to mandibular retrusion, characterized by a hypodivergent growth pattern and a normal gonial angle. Dental findings included increased proclination of the mandibular incisors and a significant increase in overjet (Figure 2).

**Figure 3:**
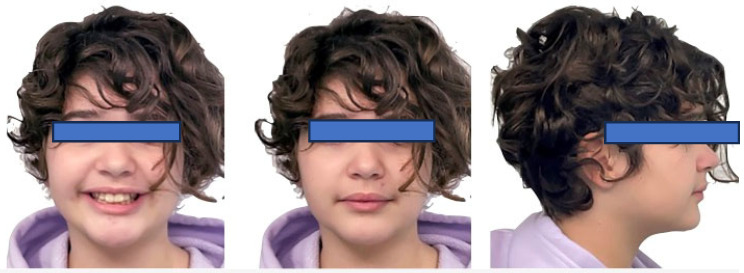
Initial photos of the patient in frontal and lateral view.

Functional examination revealed no signs or symptoms of temporomandibular disorders (TMD), and the patient reported no parafunctional habits. Upon extraoral examination, the frontal view showed facial symmetry. Profile analysis confirmed a hypodivergent facial biotype characterized by mandibular retrusion, a normal nasolabial angle, and balanced lip projection (Figure 3).

After performing the cephalometric tracing with the software *webceph* by a single examiner, it was clear that the patient has a Class II skeletal condition due to mandibular retrusion; hypodivergence, normal gonial angle, increased lower incisor inclination, and increased overjet (Figure 2).

**Figure 4:**
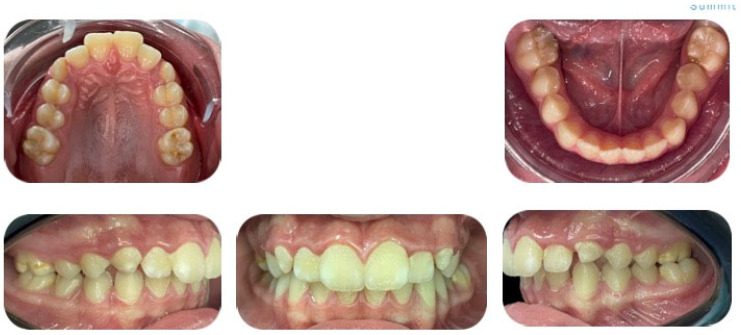
Initial intraoral photos—upper arch, lower arch and right occlusal, left occlusal.

An intraoral occlusion examination revealed:

- Class II molar and canine malocclusion.
- Upper dental midline deviated 0.5 mm to the left.
- Mild crowding in both the upper and lower arches.
- Increased overjet (more than 3 mm) and normal overbite.
- Normal Bolton index.
- Absence of ectopic teeth, rotations, malpositions, etc.
- Left posterior underbite (2.6/3.6) (Figure 4).

## 3. Treatment Planning

**Figure 5:**
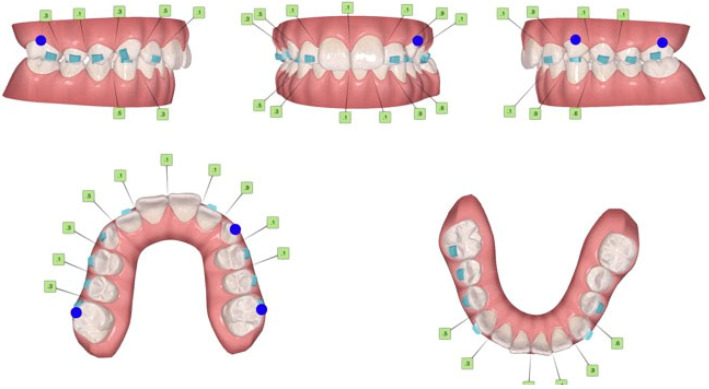
ClinCheck^®^ Invisalign system in the PRE-MA phase (PRE—mandibular advancement).

The software used for digital planning of the treatment plan was the ClinCheck^®^ Invisalign system. The protocol was strategically divided into three distinct stages: a pre-MA phase (arch preparation), the MA phase (mandibular advancement), and a final occlusal finishing phase. The specific clinical requirements (prescription) for the initial stages were as follows (Figure 5):

### Arch Preparation (Pre-MA Phase)

The primary objective of this phase was to maximize the sagittal inter-arch space (overjet space) and eliminate any occlusal interferences that could hinder mandibular advancement.

*Maxillary Arch:* Dentoalveolar expansion was performed to coordinate the arch form. To increase the available overjet, the maxillary incisors were intruded and their labial inclination was increased (using the inclination of tooth 2.1 as a reference limit).

*Mandibular Arch:* In addition to arch-form regularization, the protocol focused on reducing the labial proclination (retroclination) and intruding the mandibular incisors.

*Transverse Correction:* To resolve the left posterior crossbite, a lingual tipping (coronolingual inclination) of tooth 3.6 and a buccal tipping (coronobuccal inclination) of tooth 2.6 were requested.

*Attachment Strategy:* Due to reduced clinical crown lengths, optimized attachments could not be utilized. Instead, conventional rectangular attachments were applied to ensure adequate aligner retention.

**Figure 6:**
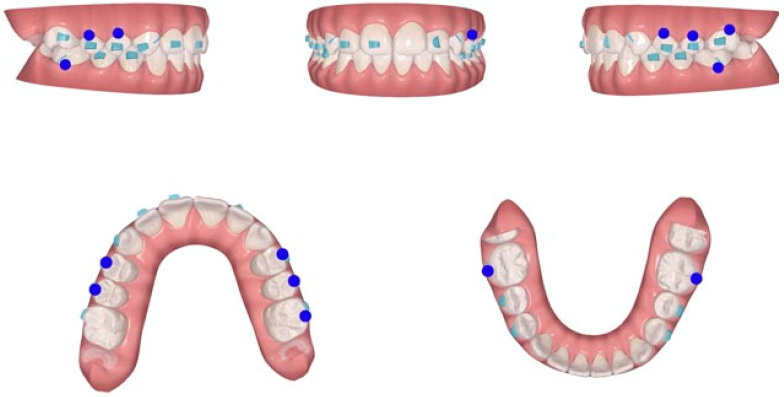
Invisalign system ClinCheck^®^ at the end of treatment.

*Mandibular Advancement Phase (MA).* The advancement was planned to achieve an end-to-end incisor relationship as the final configuration, utilizing incremental jumps of 2 mm. A specific constraint was set to avoid any mandibular dental movements aimed at midline correction during this phase, prioritizing the skeletal advancement effect (Figure 6).

*Clinical Re-evaluation and Refinement.* At stage #60, a progress scan was performed to assess the clinical expression of the planned movements. A discrepancy was observed between the digital model and the clinical results (using Progress Assessment tool in Itero), likely due to incisal premature contacts that inhibited full mandibular advancement. Consequently, a series of additional aligners (refinement) was planned, specifically requesting a 5° increase in the labial crown inclination of the maxillary incisors to further clear the sagittal space.

**Figure 7:**
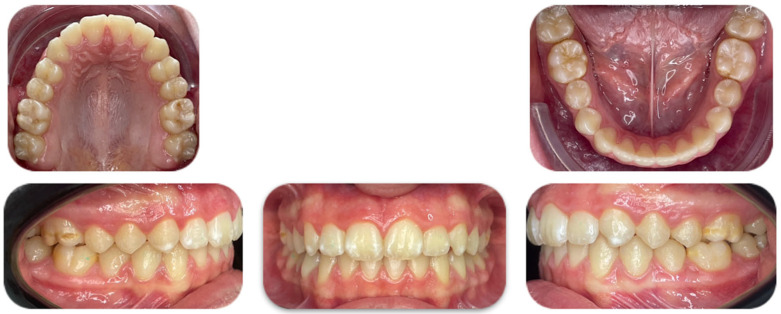
Final intraoral photos—upper arch, lower arch and right occlusal, left occlusal.

**Figure 8:**
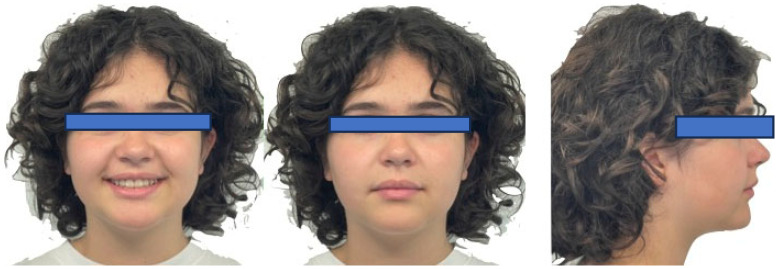
Final photos of the patient in frontal and lateral view.

The final result after two years of treatment shows the following objectives achieved (Figure 7 and Figure 8):

- First class molar, premolar and canine relationship.
- Centered midline.
- Ovb (overbite) and Ovj (overjet) are normal.

**Figure 9:**
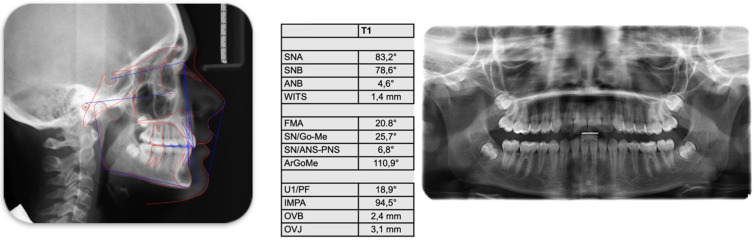
Final panoramic x-ray and final teleradiography and cephalometric analysis.

From the final cephalometric examination and from orthopantomography, the patient’s clinical conditions will be as follows (Figure 9).

- Skeletal Class I.
- Hypodivergent.
- Normal gonial angle.
- Normal lower incisor inclination.
- Normal overjet.
- Periodontal Conditions: Within normal.
- Carious Processes: Absent.
- MIH on 1.6 and 2.6.
- Fracture of the mesial portion of the incisal edge of tooth 2.1.
- Dental Formula: Permanent dentition.

## 4. Discussion

As highlighted by Proffit et al. [8], incisor position is the cornerstone of orthodontic-surgical and functional therapy, as it guides orthodontic decompensation and influences mandibular projection.

Cephalometric measurement of the spino-incisal (110° ± 5°) and mandibulo-incisal (90° ± 5°) angles is considered essential to avoid inappropriate compensation that may alter facial harmony or post-treatment stability. Two key aspects must be considered when using the Invisalign system: patient selection (i.e., treatment eligibility) and the criteria used to generate the pre-mandibular advancement (pre-MA) phase. With regard to clinical eligibility, the teeth supporting the Precision Wings must be present and stable throughout the advancement phase, and the dentition should be fully erupted, except for canines and second and third molars. No buccal supernumerary teeth should be present in the premolar–molar regions. The technique is recommended at the clinician’s discretion in cases of severe deep bite (>7 mm), in younger patients (<10 years) with residual deciduous dentition in the wing area, and in older patients (>17 years) with limited growth potential. Posterior crossbite may require correction during the pre-MA phase to avoid interference with wing positioning; incomplete correction may result in posterior occlusal interference and posterior open bite. The pre-MA phase is automatically generated under specific conditions to optimize Precision Wing positioning, including deep bite >7 mm, molar rotation >20°, initial advancement of Class II Division 2 incisors to achieve an overjet of 2 mm, and crossbite correction to obtain a posterior overjet between −2 mm and 4 mm. In both patient selection and initiation of the pre-MA phase, the software primarily considers the wearability of the Precision Wings, while diagnostic parameters are not directly incorporated, as diagnosis remains the responsibility of the orthodontist. The review by Lione et al. [4] emphasizes that the early use of aligners in mixed dentition can guide mandibular growth and promote the functional correction of Class II malocclusions, provided that the clinician carefully considers the growth phase, the projection of the lower incisors, and the vertical and sagittal relationships. The authors recommend integrating digital planning with a personalized cephalometric analysis to identify the target position of the lower incisor relative to the NB and pogonion points [9]. Similarly, Inchingolo [6] emphasizes that in aligners used for Class II children, the effectiveness of mandibular movement depends largely on the correct control of the inclination of the lower incisors, which must advance in a coordinated manner with the mandibular projection but without exceeding the alveolar limits [9]. From a methodological standpoint, the review by Turner [10] argues that the integration of comprehensive digital workflows—including intraoral scans, 3D analysis, and CAD/CAM systems—increases diagnostic accuracy and therapeutic predictability by up to ±0.2 mm. However, the article reiterates that the final clinical value depends on the practitioner’s ability to interpret the digital data with a solid understanding of the cephalometric parameters and biological limits. Systematic reviews of aligner effectiveness and clinical outcomes indicate that aligner biomechanics and auxiliaries materially influence treatment predictability, particularly in complex sagittal corrections [11]. Finally, the study by Badiali et al. [12] on virtual orthodontic-surgical planning demonstrated the usefulness of three-dimensional cephalometric analyses (3DCA) in assessing the predictability of skeletal and incisal movements. Even in complex surgical contexts, the use of 3D cephalometry has proven essential for correlating mandibular advancement with the virtually predicted incisal position. In summary, a correct diagnosis and correct digital planning, as reported in the following case report, are essential. Planning the pre-MA phase with the aim of creating the conditions for the overjet space (the inter-arch space on the sagittal plane), useful for mandibular advancement, to be as large as possible is an essential element for achieving the maximum orthopedic effect from the MA feature. Furthermore, a gradual advancement of the mandible has the dual purpose of improving the patient’s comfort and at the same time promoting the adaptation of the muscular sling to the mandibular advancement.

## 5. Conclusions

This case report outlines an optimized clinical strategy for treating mandibular retrusion in growing patients:

*External Diagnostic Integration:* Initial cephalometric analysis must be conducted outside the digital planning software to define the ideal projection of the lower incisors relative to the IMPA and NB angles. *Alveolar Respect:* Radiographic overlays should be used to ensure dental movements remain within biological alveolar limits. *Protocol Optimization:* The pre-MA phase should prioritize creating maximum overjet to facilitate greater skeletal advancement. *Incremental Progress:* Mandibular advancement should be performed in increments (≤2 mm) with periodic clinical reassessments of the functional occlusion and muscular adaptation. While this protocol offers a high degree of predictability, its success remains dependent on the timing of intervention and initial clinical conditions. The future of digital orthodontics lies in the development of predictive models capable of simulating mandibular evolution over time and automatically adjusting incisal positioning based on a patient’s residual growth potential.

## Data Availability

The original contributions presented in this study are included in the article. Further inquiries can be directed to the corresponding author.
